# Disparities in antiretroviral therapy adherence among selected adult subpopulations with HIV/AIDS in Tanzania: Analysis of a population-based survey, 2022/23

**DOI:** 10.1371/journal.pgph.0002374

**Published:** 2025-08-14

**Authors:** Dayani Adam, Ramkumar T. Balan

**Affiliations:** 1 Department of Social Sciences and Humanities, Mzumbe University – Mbeya Campus College, Mbeya, Tanzania; 2 Department of Mathematics and Statistics, University of Dodoma, Dodoma, Tanzania; Moi University College of Health Sciences, KENYA

## Abstract

Adherence is a critical component of successful HIV treatment among people living with HIV/AIDS. Yet, there is improvement in the level of adherence, but disparities are exhibited among subpopulations of adults living with HIV/AIDS. The objective of this study was to assess the disparity of the treatment adherence of selected subpopulations among HIV adult patients in Tanzania. We used a cross-sectional survey of 1427 adults living with HIV/AIDS from 31 regions in Tanzania. A two-stage stratified cluster design was used in this survey and included jackknife replicate sampling weights. Frequency tables displaying frequencies, weighted proportions, and confidence intervals were employed to depict socio-demographics by treatment adherence. Treatment adherence was the outcome variable, defined as a binary response that categorized self-reported adherence to ART at the 95% threshold: (1) “Missed 0 or 1 day,” which described high adherence, and (0) “Missed 2 or more days,” which showed low adherence. Rao-Scott chi-square was used to produce the weighted proportions of the adherence within subpopulations. Also, we used the Adjusted Wald Test to examine whether or not the subpopulations are similar to or different in terms of their level of treatment adherence. The results showed a significant difference in treatment adherence between the selected subpopulations. The majority of the chosen subpopulations, including those based on age, sex, marital status, and level of education, were found to have disparities in the level of treatment adherence. However, the study also revealed no disparities in treatment adherence between employed and unemployed HIV patients, divorced and widowed, age groups of 35–44, and those of 45 and 54. The study’s findings revealed considerable disparities in treatment adherence between various subpopulations. These findings emphasize the necessity for specifically developed interventions to address these disparities to attain better health outcomes among adults living with HIV/AIDS.

## Introduction

The Human Immunodeficiency Virus (HIV) had a profound global impact since the 1980s [1–3]. The disease has emerged as a global health crisis, rapidly spreading and impacting a vast population. As reported by [4], the disease has infected 79.3 million people globally, leading to the death of 36.3 million people since its outbreak. By the end of 2023, Acquired Immune Deficiency Syndrome (AIDS) resulted in nearly 630,000 deaths, alongside 1.3 million new HIV infections [5]. Among the 39.9 million people living with HIV (PLHIV), 38.6 million are adults, with 1.4 million being young children aged 0–14 years [5,6]. In Tanzania, a total of 1.7 million people are currently infected with 32,000 newly reported cases, and the number of deaths has reached 20,000 [7].

Recognizing the widespread impact and recurrence of HIV/AIDS infections, organizations like the Joint United Nations Programme on HIV/AIDS (UNAIDS) prioritize treatment programs. That is, the diagnosis of new HIV infections necessitates the intensification of the use of antiretroviral therapy (ART) for good health and well-being as one of the sustainable development goals and strategies to end AIDS by 2030 [8]. This aligns with a strong emphasis on HIV patients’ consistent adherence to ART, highlighting the vital need for precise timing, correct dosage, and appropriate administration of medical advice [7].

Adherence to ART has been an essential strategy for clinical success, especially viral suppression and cluster differentiation 4 (CD4) cell counts among PLHIV. In a similar vein, keeping adherence rates above the UNAIDS threshold (>95%) is critical to lowering viral loads, stopping the transmission of new infections, and subsequently reducing morbidity and mortality rates among PLHIV [9,10]. Despite substantial funding and support from organizations like the President’s Emergency Plan for AIDS Relief (PEPFAR) for ART programs in sub-Saharan countries, it has been found that adherence to ART, along with viral loads and new infection rates, remains unsatisfactory [11]. Notably, adherence to ART varies across different subpopulations, with some demonstrating high levels while others exhibit lower adherence [12].

Previous studies have reported that disparities in treatment adherence exist among various subpopulations. Most of the disparities were identified in sex, age, marital status, employment status, and residential settings. For instance, [13,14] showed that females had lower adherence than males receiving ART. These studies indicate that there is a disparity in treatment adherence among the sexes of adult patients. Also, [15] reported that participants with older ages have higher adherence compared to those with younger age. The disparity is commonly also found in the residential settings, which identified that the patients who lived in urban areas had higher adherence compared to the patients who lived in rural areas [16,17]. The other study revealed that married patients had more adherent compared to those with single and separated [18]. Additionally, disparities in treatment adherence may vary across different medical conditions and treatments. Knowing disparities may help to improve intervention. Therefore, this study aimed to determine and assess the disparity of treatment adherence of the ART of the selected subpopulations among adults living with HIV/AIDS.

## Materials and methods

### Study design, setting, and population

We conducted an analysis using cross-sectional survey data extracted from the Tanzania HIV Impact Survey (THIS) 2022/23, which collected information across 31 regions including both Tanzania Mainland and Zanzibar. THIS was a survey in Tanzania under the broader initiative known as the Population-based HIV Assessment (PHIA) [19]. The predominant goal of the PHIA project was to assess HIV prevalence and incidence in regions grappling with a high burden of HIV. The initiative received funding from PEPFAR through the US Centers for Disease Control and Prevention (CDC). Furthermore, our study focused on the population of HIV-infected adult patients aged 15 and older who were undergoing ART care.

### Sample, sample design, and sampling weights

The sample size for this study was 1427 HIV adult patients who were on ART care extracted from 33,663 participants. There were 42.7% males and 57.3% females among the participants. According to HIV testing, 5.5% (1,850) tested positive for the infection, whereas 94.5% (31,813) tested negative. ART was used by 84.05% (1,555) of patients who tested positive, and 1,427 of them reported missing doses. We adopted a two-stage stratified cluster sampling from THIS. In this sampling design, 31 regions of Tanzania were divided into 28 strata. The 26 regions of Tanzania Mainland were treated as separate strata. In the case of Zanzibar, all five regions were combined into two strata, Pemba and Unguja, where Pemba comprises Kaskazini Pemba and Kusini Pemba, and Unguja comprises Kusini Unguja, Mjini-Magharibi, and Kaskazini Unguja. In the first stage, enumeration areas formed clusters regarded as primary sampling units (PSUs) that made up 567. PSUs were selected using probabilities proportionate to the number of households based on the denominator of the 2022 population census. In the second stage, a random systematic sample was employed at rates ensuring self-weighting to select households from the initial stage of sampling. We integrated both initial and replicate jackknife sampling weights into the analysis, examining replicate weights for inclusion based on their origin from the interview or biomarker dataset. This comprehensive set, comprising the initial jackknife weight and 277 replicate weights, ensures an accurate representation of the target population by accounting for varying probabilities of selection at each sampling stage. Hence, by accounting for the complex sampling design and predicting sampling errors, jackknife replicate weights are used to get unbiased results. The details on the sampling were provided in the PHIA data use manual [20].

### Study variables

Treatment adherence was an outcome variable, calculated based on how frequently patients missed prescribed doses in the previous 30 days. Treatment adherence was a binary response that categorized self-reported adherence to ART at the 95% threshold: (1) “Missed 0 or 1 day,” which described high adherence, and (0) “Missed 2 or more days,” which showed low adherence. The predictor variables of our study are sex, age, marital status, education level, residential settings, and employment status. These variables were regarded as subpopulations.

### Data extraction

The 2022–23 THIS household dataset was downloaded in Stata format with permission from the PHIA project website. After understanding the detailed datasets and coding, further data encoding was carried out to modify variables, clean data, and align it with our study. The data encoding process aimed to ensure accuracy and reliability, enhancing the dataset’s suitability for analysis and interpretation. We obtained the PHIA datasets from https://phia-data.icap.columbia.edu/datasets?country_id=10&year_id=2022.

### Data analysis

Stata version 18 and Microsoft Excel were used in the analysis of the survey. The complex survey’s data were declared using the “**svyset**” command. This enabled integration of the two-stage stratified cluster design features in the Stata software to allow data to be analyzed as survey data. The incorporation of the “**svy**” utilized a complex survey design in each of the analyses. Also, we used “**test**” as a post-estimation command, and the disparity in treatment adherence rates among particular subpopulations was examined. Our study employed descriptive statistics, including frequencies, tables, percentages, graphs, weighted proportions, and confidence intervals, to illustrate participant characteristics about HIV and adherence status. We employed the Rao-Scott chi-square to assess the association between adherence levels and socio-demographic characteristics. Multivariable logistic regression analysis was employed to identify associations between subpopulations and treatment adherence. Variables with a P value <0.2 in bivariate analysis were identified for possible inclusion in the multivariable logistic regression analysis. Simultaneously, matched pairs were created to examine disparities in treatment adherence using the adjusted Wald statistical test. This procedure involved testing the overall equality adherence, as well as matched pairs of sex, age, marital status, residential settings, employment status, and level of education. P-values below 0.05 were regarded as statistically significant. Researchers can replicate our findings by accessing the dataset from the PHIA website and adhering to the outlined protocol. This access may require permission from the PHIA project team. It is essential to review the dataset documentation, understand its structure, and comply with data usage agreements and ethical considerations.

### Ethical consideration

The anonymized data for this study were collected from the open-access PHIA website after getting the necessary approval from the PHIA Data Manager. This extraction was performed in a manner that guaranteed privacy and confidentiality by abstaining from accessing personally identifiable information, such as file numbers, which is in line with ethical standards. The original PHIA survey protocols, including permission forms, screening forms, refusal forms, referral forms, recruitment materials, and questionnaires, were thoroughly reviewed and approved by the institutional review boards (IRBs) of Columbia University Medical Center, Westat, the CDC, the National Institute for Medical Research (NIMR), and the Zanzibar Medical Research and Ethics Committee. Such approval ensured that the collection of data through PHIA surveys was ethically conducted and that the rights and welfare of the participants were well-protected. A determination of non-human subjects’ research was obtained for our study, which involved secondary analysis of de-identified and risk-mitigated data from the PHIA surveys. This determination is a reflection that the study used anonymized data and did not interact with participants. The secondary analysis was performed by ethical guidelines for handling and analyzing de-identified data, further ensuring the confidentiality and security of participant information.

## Results

### Prevalence of treatment adherence to antiretroviral therapy among HIV adult patients

**Fig 1:**
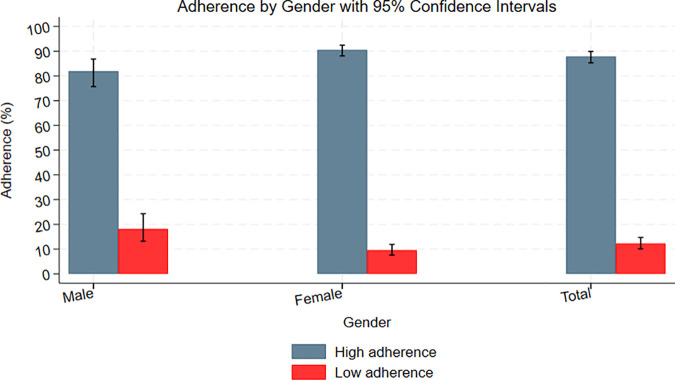
Adherence by gender.

The results revealed that 1,270 (89.0%) patients had never missed any dose or had one day missed dose, and 157 (11.0%) had two or more missed doses. HIV adult patients who had never missed any dose or had one day missed dose had adherence of 87.8% (95% CI; 85.3-89.9) whereas those two or more missed doses had low adherence about 12.2% (95% CI:10.1-14.7). Furthermore, among male HIV patients receiving ART, adherence was highest at 81.9% (95% CI: 75.7–86.8), while lower adherence was observed at 18.1% (95% CI: 13.2–24.3). Among female HIV patients receiving ART, 90.5% (95% CI: 88.1–92.4) demonstrated higher adherence, while 9.5% (95% CI: 7.6–11.9) were classified as having lower adherence. Fig 1 provides an information summary.

### Association of socio-demographic factors with treatment adherence to antiretroviral therapy

The results from the Rao-Scott chi-square test found that only gender had a statistically significant association with the level of adherence in the bivariate analysis. Also, the statistics above produced weighted proportions of adherence among all socio-demographic subpopulations. The results show that male and female adherence rates were 81.9% (95% CI: 75.7-86.8) and 90.5% (95% CI: 88.1-92.3), respectively. The highest prevalence of adherence is 91.0% (95% CI: 85.1-94.7) among the widowed. The patients without education had a high prevalence rate of 89.7% (95% CI; 84.3-93.4) compared to others. In rural settings, the prevalence was 89.4% (95% CI: 85.9-92.0). This rate runs contrary to the urban adherence rate of 85.7% (95% CI: 81.8-88.8). For age, the HIV adult patients aged 65 years and above revealed that the adherence rate is 89.8% (95% 79.3-95.3). Employed HIV adult patients show higher adherence of 88.9% (95% CI: 85.9- 91.5). The prevalence of treatment adherence to ART on socio-demographic factors is summarized in Table 1.

**Table 1:** Association of socio-demographic factors with treatment adherence to antiretroviral therapy.

Variable	High Adherence (n = 1270)		Low Adherence (n = 157)		P-value
	n	Weighted (95% CI)	n	Weighted (95% CI)	
**Gender**					0.001
Male	348	81.9 (75.7–86.8)	61	18.1 (13.2–24.3)	
Female	922	90.5 (88.1–92.3)	96	9.5 (7.6–11.9)	
**Education**					0.089
No Education	212	89.7 (84.3–93.4)	25	10.3 (6.6–15.7)	
Primary	926	88.9 (85.7–91.5)	97	11.0 (8.5–14.3)	
Secondary	121	80.0 (71.5–91.5)	33	20.0 (13.6–28.5)	
More Than Secondary	9	78.4 (19.5–98.2)	1	21.6 (1.8–80.5)	
**Marital Status**					0.303
Never Married	109	84.4 (69.8–92.7)	16	15.6 (7.3–30.2)	
Married/living together	656	88.8 (85.7–91.2)	79	11.2 (8.8–14.2)	
Divorced	254	84.5 (78.4–89.1)	41	15.5 (10.9–21.6)	
Widowed	249	91.0 (85.1–94.7)	21	9.0 (5.3–14.9)	
**Residential Setting**					0.11
Urban	424	85.7 (81.8–88.8)	68	14.3 (11.2–18.2)	
Rural	846	89.4 (85.9–92.0)	89	10.6 (7.9–14.1)	
**Age**					0.09
15–24	50	73.2 (52.1–87.2)	12	26.8 (12.8–47.9)	
25–34	214	88.9 (83.7–92.7)	25	11.0 (7.3–16.3)	
35–44	352	88.9 (84.6–92.1)	47	11.1 (7.9–15.4)	
45–54	384	87.7 (83.0–91.2)	45	12.3 (8.8–17.0)	
55–64	178	89.4 (83.1–93.5)	19	10.6 (6.5–16.9)	
65+	92	89.8 (79.3–95.3)	9	10.2 (4.7–20.7)	
**Employment**					0.29
Employed	596	88.9 (85.9–91.5)	72	11.0 (8.5–14.1)	
Not Employed	674	86.8 (82.9–89.8)	85	13.2 (10.2–17.0)	

### Multivariable binary logistic regression analysis of socio-demographic factors influencing treatment adherence to antiretroviral therapy

The multivariable binary logistic regression analysis in Table 2 revealed that gender was the only significant socio-demographic factor associated with treatment adherence to ART. Female patients had significantly higher odds of adhering to ART compared to male patients, with an Adjusted Odds Ratio (AOR) of 2.173 (95% CI: 1.432–3.298, p = 0.001).This indicates that female patients had more than twice the odds of adhering to ART compared to their male counterparts. On the other hand, other socio-demographic factors such as age, residential setting, and education level were found to be insignificant in determining ART adherence.

**Table 2:** Multivariable binary logistic regression analysis of socio-demographic factors influencing treatment adherence to antiretroviral therapy.

Variable	AOR95% CI	Jackknife Std.Error	P-value
**Sex (Ref = Male)**			
Female	2.173 (1.432,3.298)	0.44	0.001
**Age (Ref = 15–24)**			
25–34	2.319 (0.904,5.944)	1.06	0.078
35–44	2.457 (0.918,6.573)	1.174	0.072
45–54	2.193 (0.827,5.819)	1.039	0.11
55–64	2.736 (0.918,8.158)	1.451	0.069
65 and above	2.966 (0.83,10.602)	1.835	0.091
**Residential Setting (Ref = Urban)**			
Rural	1.313 (0.853,2.021)	0.275	0.206
**Education (Ref = No education)**			
Primary	1.059 (0.582,1.926)	0.308	0.846
Secondary	0.622 (0.299,1.294)	0.221	0.194
More than secondary	0.640 (0.243,1.684)	0.301	0.351

### Disparity of treatment adherence to antiretroviral therapy among sub-population

The study found that the treatment adherence rate was 87.8%, significantly lower than the desired level of 95% (F = 41.46, p < 0.001). Adherence differed significantly between males and females (F = 234.14, p < 0.001). Substantial disparities were also noted in terms of age, with younger patients, aged 15–24 years, showing lower adherence than the rest. However, no differences were found across groups of patients who were 35–44 and 45–54 years old (F = 0.40, p = 0.533). The urban setting demonstrated disparities in adherence with the rural setting (F = 13.95, p = 0.001). Employment status was not significantly related to adherence (F = 0.70, p = 0.4122). Table 3 provides the summary of the disparities of treatment adherence to ART among Sub-populations.

**Table 3:** Disparities of treatment adherence to antiretroviral therapy among sub-population.

Matched Pair		Adjusted Wald Statistic	P-value	Status
**Sex**				
Female	Male	F = 234.14	<0.001	**✓**
**Age**				
15–24	25–34	F = 59.59	<0.0001	**✓**
15–24	35–44	F = 246.36	<0.0001	**✓**
15–24	45–54	F = 196.95	<0.0001	**✓**
15–24	55–64	F = 46.83	<0.0001	**✓**
15–24	65+	F = 6.80	0.0151	**✓**
25–34	35–44	F = 22.01	<0.0001	**✓**
25–34	45–54	F = 27.32	<0.025	**✓**
25–34	55–64	F = 6.30	0.0189	**✓**
25–34	65+	F = 39.10	<0.0001	**✓**
35–44	45–54	F = 0.40	0.533	**X**
35–44	55–64	F = 61.72	0.0002	**✓**
35–44	65+	F = 135.68	<0.0001	**✓**
45–54	55–64	F = 67.51	<0.0001	**✓**
45–54	65+	F = 147.84	0.0001	**✓**
55–64	65+	F = 19.13	0.0002	**✓**
**Residential Settings**				
Urban	Rural	F = 13.95	0.001	**✓**
**Education Level**				
No Education	Primary	F = 295.76	<0.0001	**✓**
No Education	Secondary	F = 7.32	0.0120	**✓**
No Education	More than Secondary	F = 123.14	<0.0001	**✓**
Primary	Secondary	F = 329.86	<0.0001	**✓**
Primary	More than Secondary	F = 942.25	<0.0001	**✓**
Secondary	More than Secondary	F = 53.67	<0.0001	**✓**
**Marital Status**				
Never	Married	F = 270.04	<0.0001	**✓**
Never	Divorced	F = 35.51	<0.0001	**✓**
Never	Widowed	F = 19.30	<0.0001	**✓**
Married	Divorced	F = 122.90	<0.0001	**✓**
Married	Widowed	F = 133.35	<0.0001	**✓**
Divorced	Widowed	F = 1.01	0.3245	**X**
**Employment Status**				
Not Employed	Employed	F = 0.70	0.4122	**X**

**✓ =** Statistically significantly different, **X** = Not Statistically significantly different.

## Discussions

The results of the current study show that there are disparities in adherence to ART in the selected subpopulations of HIV adult patients. These disparities reveal variations in adherence within subpopulations, suggesting that some may experience high adherence while others exhibit low adherence. Such a situation is potentially linked to the use of different antiretroviral regimens. The identification of these disparities highlights a need for targeted interventions and personalized healthcare approaches to address varying factors influencing treatment adherence within these groups. Other similar studies have also reported existing disparities between adherence and demographic characteristics [21,22].

We identified that an overall treatment adherence prevalence statistically falls behind the recommended level of 95%. This finding signifies the failure to achieve optimal adherence to ART in HIV adult patients through current interventions. This aligns with previous studies reporting similarly low adherence rates [18,23]. However, inconsistencies arise with studies reporting higher adherence rates than the UNAIDS recommendation [24–27]. Despite the overall sub-optimal adherence, notable variations persist across subpopulations, with adherence levels ranging from low to high, often reflecting differences in the methods used to assess adherence.

Our study found that disparities persist in different subpopulations. For instance, we found that females demonstrated higher adherence to ART compared to males. This disparity was statistically significant and suggests that women may be more consistent in following treatment regimens, possibly due to greater health-seeking behavior, stronger social support, and regular contact with healthcare services. In contrast, men may face more challenges, such as stigma, work-related constraints, or cultural norms that limit their engagement with healthcare systems. This result aligns with studies by [13,14,23]. The other studies found no statistically significant differences between male and female adherence rates [28,29].

Also, Adherence to ART improved progressively with age. Younger adults, particularly those in the youngest age group, showed the lowest adherence, while older individuals exhibited consistently higher adherence. This may be due to the stability and routine that often come with age, along with increased health awareness and life experience. Conversely, younger individuals may encounter challenges such as stigma, lifestyle disruptions, or denial of their condition, all of which can negatively impact adherence. The other studies align with the current findings which show significant differences in adherence rates across age subgroups [15]. However, our results contrasted with [30,31] who found no statistically significant variations in adherence rates between older and younger individuals.

Interestingly, individuals with no formal education demonstrated higher adherence than those with secondary or post-secondary education. This pattern was unexpected and suggests that adherence is not necessarily improved by higher education. Those without formal education may be more likely to trust and follow healthcare provider instructions strictly, while those with more education may question treatment protocols, experience busier schedules, or manage competing life demands that hinder adherence. This observation aligns with a study by [32] in a nationwide retrospective cohort study in Finland. Notably, these findings diverge from studies conducted which suggested that the level of education does not exhibit statistical differences in terms of adherence between different groups [29,30,33].

Among marital status categories, widowed and married individuals exhibited higher adherence than those who were never married or divorced. This suggests that emotional and social support, particularly from a spouse or family, may play a significant role in maintaining adherence. Individuals who are never married or divorced might lack such support systems, leading to increased vulnerability to non-adherence. This finding reinforces the importance of psychosocial support in adherence interventions. This result aligns with studies that showed that married HIV adult patients had a significant disparity in treatment adherence compared to unmarried and widowed HIV adult patients [18,34–36]. This finding was contrary to the study by [37] in Ethiopia who asserted that only married appeared to have a disparity in adherence and widowed.

In this study, participants residing in rural areas showed better adherence compared to their urban counterparts. This finding challenges the common assumption that urban residents, due to better access to health facilities, are more adherent. Rural patients may benefit from stronger community networks, closer monitoring by community health workers, or more stable lifestyles. On the other hand, urban patients may face fragmented care, stigma, and social isolation, which could negatively affect adherence. This research supported the studies, which found that there were significant differences in adherence rates between rural and urban residents [17,38,39]. This consistency supports the notion that geographical location alone may not be a decisive factor in predicting treatment adherence. These findings are contrary to the findings of those who reported that there were disparities in adherence in urban and rural settings [29,40,41].

Contrary to expectations, in this study, we found that there is no significant difference in adherence was observed between employed and unemployed participants. This suggests that employment status, in isolation, may not be a strong predictor of adherence. While employment might provide the financial means to support treatment, it can also introduce time and stress-related constraints. Unemployed individuals may face economic challenges but might have more time and flexibility to engage with treatment protocols. These counterbalancing factors likely explain the similarity in adherence levels. This research supported the studies, which found that there were no significant differences in adherence rates between employed and unemployed HIV patients [29,42]. This consistency supports the notion that employment status alone may not be a decisive factor in predicting treatment adherence. These findings are contrary to the findings of those who reported that there were disparities in adherence in employed and unemployed HIV patients [43].

Based on the findings of this study, we recommend addressing disparities in ART adherence through tailored interventions aimed at determining specific adherence factors within each subpopulation. This personalized healthcare approach aims to optimize treatment outcomes, fostering more equitable health outcomes. We also recommend the implementation of continuous adherence monitoring mechanisms to facilitate timely identification and proactive intervention. Furthermore, a comprehensive understanding of contextual factors, including demographics and geography, is essential for addressing disparities in adherence. These factors enable the development of nuanced and effective interventions tailored to the diverse needs of populations.

### Strengths and limitations of the study

Our study is strengthened by the inclusion of a nationally representative sample of 1427 HIV adult patients and the application of complex survey analysis. Both strengths ensure a precise representation of disparities in treatment adherence among HIV adult patients. However, there are several limitations to the interpretation of the study’s conclusions. First off, the only method used to measure adherence was patient self-reporting of missing doses. This introduced the possibility of social desirability and recall bias, which could have led to an overestimation of adherence. Second, only persons aged 15 and older who were receiving ART during the study period could generalize the results. Thirdly, changes in individuals’ adherence to ART over time could not be completely captured by the cross-sectional approach.

## Conclusions

We have demonstrated the differences in treatment adherence across several subpopulations, including age, sex, marital status, education level, and residential settings. The variations in adherence to ART among different subgroups, as highlighted in this study, can provide valuable insights for developing programs and policies focused on mitigating these differences in health outcomes, particularly viral load suppression. Future interventions should focus on identifying the causes behind these variances in the selected subpopulations.
